# Photothermal Response of Hollow Gold Nanorods under Femtosecond Laser Irradiation

**DOI:** 10.3390/nano9050711

**Published:** 2019-05-07

**Authors:** Rongping Gan, Haihua Fan, Zhongchao Wei, Haiying Liu, Sheng Lan, Qiaofeng Dai

**Affiliations:** Guangdong Provincial Key Laboratory of Nanophotonic Functional Materials and Devices, School of Information and Optoelectronic Science and Engineering, South China Normal University, Guangzhou 510006, China; z30803080@126.com (R.G.); 20111249@m.scnu.edu.cn (H.F.); weizhongchao@263.net (Z.W.); hyliu@vip.163.com (H.L.); slan@scnu.edu.cn (S.L.)

**Keywords:** surface plasmon resonance, photothermal response, two-temperature model, femtosecond laser

## Abstract

The photothermal kinetics of hollow gold nanorod (HGNR) under femtosecond laser irradiation are studied numerically with finite-element methods and a two-temperature model. Compared with solid gold nanorod (SGNR) with the same aspect ratio (AR), the localized surface plasmon resonance (LSPR) peak of HGNR can be red-shifted to the second near-infrared window, and the absorption cross-section of HGNR can be larger than that of SGNR. In addition, under the influence of an applied numerically electromagnetic field (simulated femtosecond laser irradiation), the heat generated by HGNR makes the temperature rise of the surrounding medium faster and higher. Compared with SGNR with the same resonance wavelength, HGNR has a slightly smaller absorption cross-section but can achieve a higher temperature rise of the external medium. In addition, the laser energy, required to achieve the critical temperature for selective photothermal damage of tumor cells, is also significantly reduced. Moreover, with the same incident laser energy, the decreasing of HGNR shell thickness leads to an increase of the temperature rise of the external medium, while the change of femtosecond laser pulse width will not significantly change the temperature rise of its lattice and the external medium. In short, this study aims to provide some useful insights for the applications of HGNR in photothermal tumor therapy.

## 1. Introduction

In recent years, gold nanoparticles (GNPs) have attracted many research interests because of their special photophysical properties. This is mainly because the collective and coherent oscillations of free electrons or charge carriers of the particles driven by an external electromagnetic field generate localized surface plasmon resonance (LSPR). The characteristic frequency of LSPR ω_sp_, called the plasmon frequency, can be tuned by changing the size, shape, and structure of the particles. In the interaction with external electromagnetic fields, GNPs present unique optical and thermal properties in a specific frequency range. When the frequency of illumination is near to ω_sp_, due to the near-field enhancement of GNPs, GNPs absorb more light energy and convert it to heat [1,2]. As a result, GNPs are used as an effective nano heat source under visible or infrared illumination [3]. The photothermal effect of GNP even plays a key role in information recording [4]. In addition, GNPs have good biocompatibility, low toxicity, and controllable surface chemical properties, so that they have a broad prospect in biomedical applications [5,6]. Among them, the photothermal therapy is becoming one of the most active research directions [7,8,9].

In biomedical applications, the near-infrared (NIR) light can penetrate deeply into soft tissue and blood. In the NIR region, there are two biological transparency windows: the first NIR window with a wavelength of 650–950 nm (NIR-i window) and the second NIR window with a wavelength of 1000–1350 nm (NIR-ii window) [10]. The light in NIR-ii window generally obtains better tissue transmission through lower scattering and lower energy absorption. In the reference [11,12,13], it shows that the light in the NIR-ii window has deeper tissue penetration ability, lower self-fluorescence background, higher maximum allowable exposure value, and lower photon scattering, which is more suitable for biological applications. However, for gold nanomaterials, it is difficult to design and synthesize highly responsive gold nanostructures in the NIR-ii window due to the requirements of particle size for clinical applications. In the reference [14,15], it indicates that the nanostructures having a size greater than 200 nm are not suitable for in vivo applications, because they can be easily accumulated in organs or tissues, such as the liver and spleen, under intravenous injection. Therefore, the utilization of how gold nanoparticles responded to the NIR-ii window has not yet been well studied [16]. Most recently, a mini or ultra-thin gold nanorod was prepared with a tunable LSPR peak in the NIR-ii window [17,18], due to its improvement in size, making it a very promising biological application material. Kai Cai and his colleagues used a Se-doping Te nanorod-templated method with the assistance of L-cysteine for the first time to fabricate hollow gold nanorods (HGNRs) with controllable aspect ratio (AR), and its longitudinal LSPR peak is adjustable to the NIR-ii window at a relatively small size. In addition, because of its hollow structure, it is expected to increase its drug loading, which is a potential platform for photothermal tumor therapy [19].

In clinical applications, people hope to minimize the laser energy and the time of laser-biological action while ensuring the therapeutic effect [20]. Femtosecond laser pulses can provide high peak intensity in a short time under low pulse energy, which meets this requirement well. The sharp and short-term temperature rise of illuminated GNPs also helps to realize local fine heating and avoid heating the whole medium [21].

In this paper, based on the two-temperature model, the photothermal kinetic process of HGNR under femtosecond laser irradiation is firstly studied with finite-element methods. For comparison, two solid gold nanorods (SGNRs) with different AR are also studied. After that, we compare the energy threshold to reach the temperature required for photothermal tumor therapy. Finally, with fixed length and diameter of HGNR, the temperature changes of electrons, lattice, and the surrounding medium are also studied by changing the pulse width of femtosecond laser or the shell thickness.

## 2. Methods

The energy deposition process in GNPs under pulse laser irradiation is as follows: Firstly, the plasmon of GNPs induced by light can be damped through the creation of hot electron-hole pairs via Landau damping. The hot electrons initially possess a non-equilibrium energy distribution and will quickly redistribute their energy by electron–electron scattering [22,23]. The new thermal equilibrium of electrons with a Fermi-Dirac-like distribution is subsequently formed around 100 fs to 1 ps. After that, due to the electron–phonon scattering, the energy of the electrons is transferred to the lattice so that the temperature of the lattice begins to rise. The thermal balance between the electrons and the lattice is reached at several ps. As the temperature of the lattice rises, the energy exchange between the lattice and the surrounding medium begins to take place through phonon–phonon scattering. The temperature of the surrounding medium rises gradually. The temperature jumps at the interface between GNPs, and the surrounding medium finally disappears within several ns (depending on the size of the GNPs and the intensity of the laser pulse), and the whole system will be in a state of thermal equilibrium. Here, the "two-temperature model" is used to study the temperature variation of electron and lattice subsystem. The temperature rise in the surrounding medium is calculated by a thermal diffusion equation. The corresponding equations are as follows [24]:

(1) $$C_{e}(T_{e})\frac{\partial T_{e}(r,t)}{\partial t}=\nabla \cdot (k_{e}\nabla T_{e}(r,t))-g[T_{e}(r,t)-T_{l}(r,t)]+S(t)$$

(2) $$C_{l}(T_{l})\frac{\partial T_{l}(r,t)}{\partial t}=\nabla \cdot (k_{l}\nabla T_{l}(r,t))+g[T_{e}(r,t)-T_{l}(r,t)]$$

(3) $$\rho_{m}(r)C_{m}(r)\frac{\partial T_{m}(r,t)}{\partial t}=\nabla \cdot (k_{m}\nabla T_{m}(r,t))$$

Thermal diffusion at the interface between GNPs and the surrounding medium is given by:

(4) $$F=G(T_{l}-T_{mi}),i=\left\{\begin{array}{c} 1,\mathrm{outer}\;\mathrm{interface}\\ 2,\mathrm{inner}\;\mathrm{interface} \end{array}\right.$$

where subscript *e, l, m* denotes electrons, lattice, and the surrounding medium, respectively. *T(r,t)* is the local temperature varying with time and space, and the initial temperature of the GNPs and surrounding medium is set to 300 K in numerical calculation. *C* is the heat capacity and *k* is the thermal conductivity. *g* is the electron–lattice coupling coefficient, which describes the energy exchange from electron to lattice through electron–phonon scattering. *G* is the interface thermal conductivity between GNPs and the surrounding medium, which describes the energy transfer from the lattice to the surrounding medium. Equation (1) stands for heat equation for the electron system. On the right-hand side of Equation (1), the first term expresses electron energy dissipation via heat conduction described by Fourier’s law. The second term expresses the energy exchange between the electron and the lattice with the electron–phonon coupling coefficient of g. The third term is source term originating from the laser energy absorbed by the GNPs. Similarly, Equation (2) stands for heat equation for the lattice system. The heat diffusion in the surrounding medium is described with Equation (3). Specific calculation parameters are shown in Table 1. The expression *S(t)* can be written as:

(5) $$S(t)=\frac{C_{abs}^{\lambda }\cdot P(t)}{V_{p}}$$

(6) $$P(t)=\frac{F_{L}}{\sqrt{2\pi }t_{\sigma }}\exp({\frac{-[t-t_{0}]}{2t_{\sigma }^{2}}}^{2})$$

where *S(t)* is the laser energy absorbed by the GNPs. $C_{\mathrm{abs}}^{\lambda }$is the absorption cross-section of the GNPs and *V_p_* is the volume of the GNPs. *P(t)* is the intensity of the pulsed laser with a Gaussian distribution, and *F_L_* is the energy fluence of the incident laser. $t_{\sigma }=t_{l}/2\sqrt{2\ln2}$, in which *t_l_* is the laser pulse width (the full width at half maximum of the Gaussian temporal profile) and *t_0_* is the position of the center of the peak [25].

In the following calculation, the inner and outer mediums of HGNR are set as water, and the SGNR in water is also constructed for comparison. In reference [2], it points out that the water is enough to mimic the environment of cells in the process of laser acting. Since nanoparticles with the diameter of 40 nm are very popular in biological applications [25], the diameter of gold nanorods (GNRs) in the following calculation is fixed to 40 nm while changing the AR or the thickness of the shell of GNRs. At the same time, the maximum sizes of GNRs are guaranteed to be within the scope of in vivo applications (<200 nm). By using the finite element method provided by the commercial software COMSOL (www.comsol.com), the models were solved in three-dimensions. In addition, the electromagnetic interaction between the GNP-water system and laser pulse, as well as more detailed modeling processes, are provided in Supplementary Materials.

In the process of calculation, the absorption of incident laser in the inner core of HGNR and surrounding medium is neglected. The phase changes are not included in the model, since the lattice temperature of GNP is below its melting temperature (~1337 K) and the temperature of water is below its explosive boiling temperature (~647 K) [23] in the following calculation.

## 3. Results and Discussion

### 3.1. Longitudinal Absorption Spectra of HGNR and SGNR

In biomedical applications, the light in the near infrared region has good tissue penetration. However, for the simulated structures (HGNR or SGNR), the wavelengths of transverse plasmon modes are usually difficult to adjust to the near infrared region. Therefore, in this paper, we mainly research the longitudinal plasmon modes of the structures.

**Figure 1:**
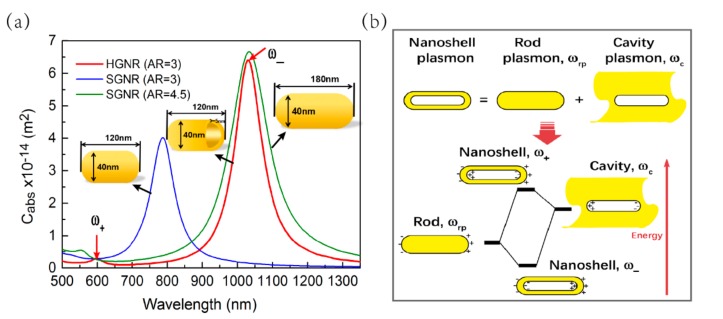
(**a**) Absorption spectra (longitudinal mode) of hollow gold nanorod (HGNR) with aspect ratio (AR) = 3 and solid gold nanorod (SGNR) with AR = 3 or 4.5. The red arrows refer to the two plasmon modes (ω_-_ and ω_+_) of HGNR. (**b**) The diagram is describing the plasmon hybridization between the rod and cavity plasmon models. The hybridization results in the splitting of the plasmon resonances of HGNR into two new resonances: the lower-energy ω_-_ "bonding" plasmon and the higher-energy ω_+_ "anti-bonding" plasmon.

Figure 1a shows the absorption cross-sections (longitudinal mode) of HGNR with AR = 3 and SGNR with AR = 3, 4.5. The maximum absorption cross-section of SGNR with AR = 3 is 3.93 × 10^−14^ m^2^ (The corresponding resonance wavelength is 784 nm occurring at the NIR-i window). While for the HGNR with the same AR, the maximum absorption cross-section is 6.32 × 10^−14^ m^2^, which is about 1.6 times greater than that of SGNR, and the corresponding resonance wavelength is moved to 1033 nm appearing in the NIR-ii window. Moreover, a small peak with a wavelength of 600 nm appears. The movement and splitting of the LSPR peak of HGNR can be attributed to the plasmons hybridization in the HGNR, that is, the interaction between the essentially fixed-frequency plasmon response of a nanorod and that of a nanocavity. As shown in Figure 1b, the interaction results in the splitting of the plasmon resonances of HGNR into two new resonances: the lower-energy *ω*_−_ "bonding" plasmon and the higher-energy *ω*_+_ "anti-bonding" plasmon. Only the lower energy *ω*_−_ "bonding" plasmon will interact strongly with the incident light field. The degree of splitting and the magnitude of the displacement of the plasmon resonances depends on the size, the shell thickness of HGNR, the surrounding environment, etc. [27,28,29].

Of course, by adjusting the AR, the LSPR peak of SGNR is also tuned to near 1033 nm. Figure 1a shows that the absorption cross-section of SGNR (AR = 4.5) at wavelength 1033 nm is 6.71 × 10^−14^ m^2^, which is a little higher than that of HGNR. However, the following calculations also indicate that the temperature rise of the surrounding medium caused by SGNR is obviously lower than that caused by HGNR. In addition, the increase of AR will increase the size of SGNR, which is not suitable for in vivo applications.

### 3.2. Photothermal Response of HGNR and SGNR under Femtosecond Laser Irradiation

**Figure 2:**
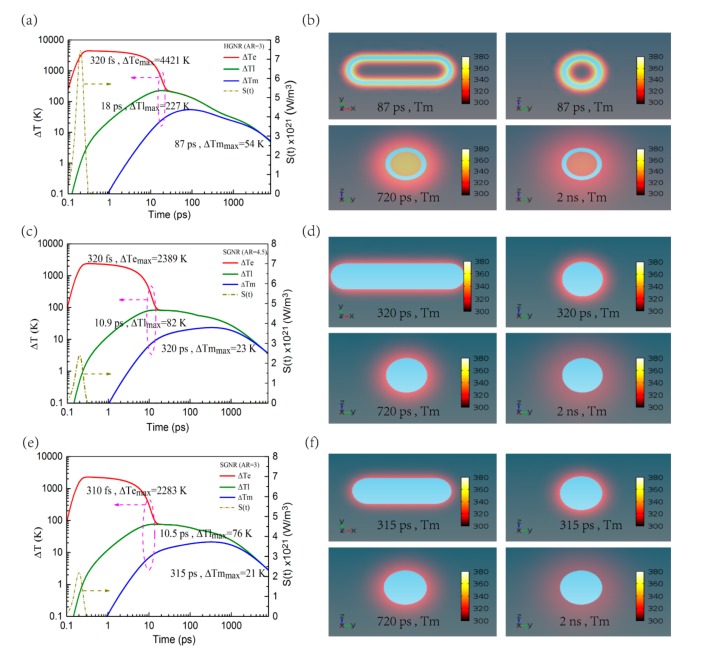
Time-dependent temperature rise are shown in (**a**), (**c**), and (**e**), in which Δ*Te*, Δ*Tl*, and Δ*Tm* are the temperature rise of electrons, lattice, and surrounding medium, respectively. Δ*Tm* is obtained at a distance of 0.3 nm from gold nanorod (GNRs). All GNRs are illuminated with a laser pulse of 100 fs and 0.8 J/m^2^, and the absorbed energy profiles are shown with the olive dotted curve. The temperature–slice maps of the surrounding medium at different times are shown in (**b**), (**d**), and (**f**). The slices cross the center along the transverse or longitudinal sections. In (**a**) and (**b**), AR = 3 for HGNR; In (**c**) and (**d**), AR = 4.5 for SGNR; In (**e**) and (**f**), AR = 3 for SGNR.

Under femtosecond laser irradiation, one common phenomenon is the heating of the GNPs and the subsequent heat transfer to the surrounding medium. When GNPs are exposed to laser pulse, the free electrons of GNPs absorb the energy of photons and become high-energy electrons, causing their temperature to rise [23]. For the HGNR, as can be seen from Figure 2a, under the irradiation of a laser pulse of 100 fs with the fluence of 0.8 J/m^2^, the temperature rise of electrons Δ*Te* rapidly reaches a maximum value of 4421 K. Subsequently, these high-energy electrons transfer energy to the lattice through electron–phonon scattering, making the lattice temperature rise by 227 K in 18 ps, while the electrons temperature gradually decreases. By phonon–phonon scattering, the lattice will also transfer energy to the water inside and outside the shell, which increases the temperature of the surrounding medium. In photothermal therapy, the thermal effects of nanoparticles on external medium are mainly concerned, and in practice they may not be directly connected to biological organs or tissues such as cell membranes, but often need to be functionalized by biochemical materials (such as antibodies, antigens, or ligands) [30]. Therefore, instead of focusing on the thermal effects of the HGNR on its internal medium, the thermal effects of it on its external medium are primarily investigated. As shown in Figure 2a, the external medium temperature rise Δ*Tm* at 0.3 nm away from HGNR reaches the maximum value of 54 K at 87 ps. Figure 2b shows some temperature-slice maps (transverse and longitudinal sections through the center) of the external medium at 87 ps, 720 ps, and 2 ns respectively, from which we visualize a thermal diffusion process in the surrounding medium. The temperature rise zone of the external medium is gradually expanding with the passage of time.

As a comparison, Figure 2c,d also show the case of SGNR with AR = 4.5 under the same irradiation condition, in which the temperature rise of its electrons, lattice, and surrounding medium are significantly small. Though the absorption cross-section of the SCNR is slightly higher than that of the HGNR, the increase of SGNR AR will increase not only its mass but also the number of electrons and lattice parts to be heated. As a result, the temperature rise of SGNR and its surrounding medium is not as high as that of HGNR with the same incident laser energy. Figure 2e,f also show the case of SGNR with AR = 3, in which the temperature rise of its electrons, lattice, and surrounding medium is also small. There are two primary reasons for it: On the one hand, the absorption cross-section of SGNR is smaller than that of HGNR, as shown in Figure 1a, making the absorbed energy of SGNR less than that of HGNR under the same incident laser energy. On the other hand, since the mass of SGNR is greater than that of HGNR, there are more electrons and lattice parts that need to be heated. Therefore, when the incident laser energy is the same, the temperature rise of the SGNR and its surrounding medium are much smaller than that of HGNR. 

In addition, in Figure 2c–f, including the temperature rise of the surrounding medium, the temperature rise of SGNR with AR = 3 and 4.5 are not much different. According to Equation (5), this is due to the very close ratio of their absorption cross-section of GNR to the volume of gold (the absolute difference value is 0.027). In other words, the SGNRs with AR = 3 and 4.5 almost absorb the same amount of energy per unit mass at the same incident laser energy, which makes the temperature rise very close.

### 3.3. Energy Threshold Required for Photothermal Destruction of Cancer Cells

**Figure 3:**
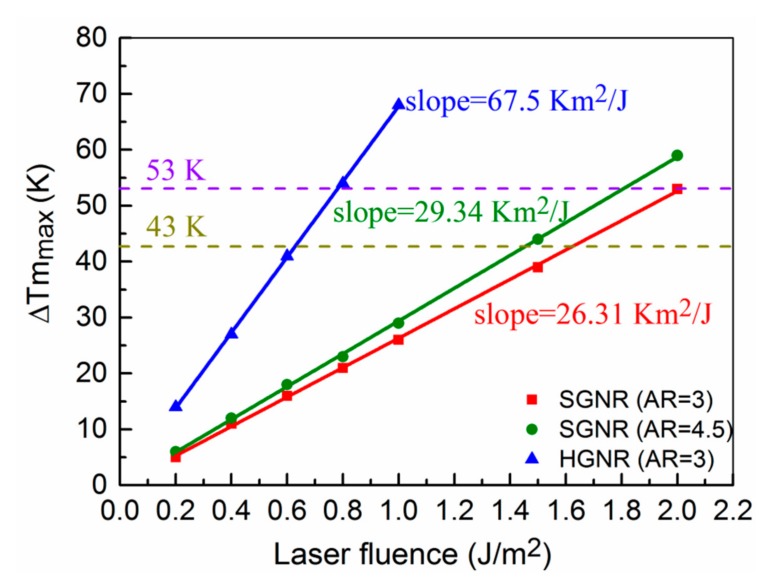
The relationship between the maximum temperature rise of the medium and the incident laser fluence. The blue, green, and red lines correspond to HGNR with AR = 3, SGNR with AR = 4.5, and SGNR with AR = 3, respectively. The sampling position of the maximum temperature rise of the medium is at a distance of 0.3 nm from GNRs. The femtosecond laser pulse width used is 100 fs.

For the above three structures, the energy thresholds of incident laser required for photothermal therapy of cancer are researched in the following. For the photothermal damage of cancer cells, the cells begin to be damaged at 70 °C and are completely destroyed at 80 °C [31]. Correspondingly, the interval of temperature rise of the surrounding medium ranges from 43 K to 53 K for the HGNR. In Figure 3, by changing the incident laser fluence, the relationship between the maximum temperature rise of the medium and the incident laser fluence is obtained. The relationship curves are linear, and the curve of HGNR has the highest slope among them. This means that the required laser fluence for the HGNR is significantly lower to reach the same temperature. In general, the lower the incident laser fluence is, the less damage the surrounding healthy tissue suffers. Therefore, from this point of view, HGNR is very beneficial to the photothermal therapy.

### 3.4. Photothermal Response of HGNR at Different Shell Thickness or Different Pulse Width

**Figure 4:**
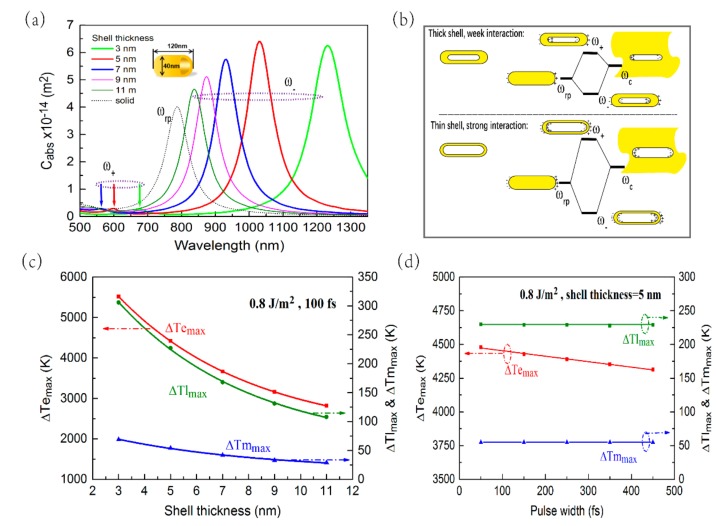
(**a**) Longitudinal absorption spectra of HGNR with different shell thickness under fixed length and diameter, in which the black dashed line is the longitudinal absorption spectra of SGNR with the same AR. The plasmon resonance peaks in the purple dashed ellipse which refers to the two plasmon modes ω_-_ and ω_+_. (**b**) The diagram of the weak and strong interaction between the pure rod plasmon and pure cavity plasmon. (**c**–**d**) the maximum temperature rise of the electrons, lattice, and the medium (at a distance of 0.3 nm from HGNR) at different shell thickness (**c**) or different femtosecond laser pulse width (**d**).

In order to further investigate the photothermal response of HGNR, we studied the influences of its shell thickness and femtosecond laser pulse width on the temperature rise of HGNR and the surrounding medium. Figure 4a shows the absorption cross-sections of HGNR with different shell thicknesses, in which the length and diameter of HGNRs are fixed (120 × 40 nm). The LSPR peaks of HGNR appear red-shifted as the shell thickness decreases. As shown in Figure 1b and Figure 4b, the pure rod plasmon induces surface charges at the outer interface, while pure cavity plasmon induces surface charges at the inner interface. The interaction strength between inner and outer charges is decided by the shell thickness. As the shell thickness decreases, the interaction strength will increase. The stronger the interaction, the further the red-shift of the "bonding" plasmon pattern [27,29]. Furthermore, as the shell thickness decreases from 11 nm to 5 nm, the absorption cross-section gradually increases, and as the shell thickness decreases from 5 nm to 3 nm, the absorption cross-section decreases slightly. Figure 4c shows a change curve of maximum temperature rise of the electrons, lattice, and the medium (at a distance of 0.3 nm from HGNR) at different shell thickness under the laser illumination of 100 fs and 0.8 J/m^2^. As the shell thickness increases, the maximum temperature rise of the HGNR and the medium gradually decreases. In Figure 4d, for fixed shell thickness (5 nm) and fixed incident laser energy fluence (0.8 J/m^2^), when the femtosecond laser pulse width increases, the maximum temperature rise of the lattice and external medium is almost independent of the femtosecond laser pulse width, but the maximum temperature rise of the electrons decreases. For the calculation in Figure 4d, the peak of the laser power will gradually decrease with the increase of the pulse width, and the entire pulse energy remains unchanged. As a result, on the one hand, since the pulse energy is first transferred to the electrons, and the electrons have a relatively small heat capacity, the electrons can respond quickly to the transient power of the pulse. That means the maximum temperature rise of the electrons will decrease with increasing pulse width. On the other hand, since the pulse energy is transferred to the lattice through the interaction between the electrons and the lattice, and the lattice has large heat capacity, the lattice has no response to the transient power of the pulse, but to the entire pulse energy. The same pulse energy determines that the maximum temperature rise of the lattice is almost the same. For the mechanism to maintain unchanged temperature rise, the surrounding medium is similar to lattice.

## 4. Conclusions

In this paper, the photothermal responses of HGNR have been numerically studied by using a two-temperature model. Compared with SGNR (AR = 3), the longitudinal LSPR peak of HGNR with the same AR can be red-shifted to the NIR-ii window (1000–1350 nm), and the absorption cross-section of HGNR increases by about 1.6 times. By increasing the AR of SGNR to 4.5, though the longitudinal LSPR peak and absorption cross-section of SGNR and HGNR can be adjusted very closely, the HGNR has a smaller size for better biocompatibility. Most importantly, under the same input laser energy, the maximum temperature rise of external medium of HGNR is obviously higher than the other two structures. Finally, we further investigated the influences of shell thickness and femtosecond laser pulse width on the photothermal response of HGNR. It is found that the maximum temperature rise of electrons, lattice, and external medium decreases with the increase of shell thickness, while the femtosecond laser pulse width has little effect on the maximum temperature rise of lattice and external medium. 

The HGNR is very beneficial to its application in photothermal tumor therapy, in which the GNRs with smaller size, the laser working at lower energy and in the NIR-ii window are required. In addition, due to the hollow structure, HGNR may also be used for drug loading, which is expected to improve the efficacy of photothermal tumor treatment.

## Figures and Tables

**Table 1:** Parameters used in numerical calculation.

Parameter	Value	Reference
**Gold properties**		
The electron heat capacity, *C_e_* (J·m^-3^·K^-1^)	70.0 × *T_e_*	[25]
Thermal conductivity of electron, *k_e_* (W·m^-1^·K^-1^)	300	[25]
The lattice heat capacity, *C_l_* (J·m^-3^·K^-1^)	3 × 10^6^	[25]
Thermal conductivity of lattice, *k_l_* (W·m^-1^·K^-1^)	0.001 × *k_e_*	[25]
Electron-lattice coupling coefficient, *g* (W·m^-3^·K^-1^)	2 × 10^16^	[25]
Dielectric function	Johnson and Christy	[26]
**Water properties**		
Density, *ρ_w_* (kg·m^-3^)	1000	[25]
Heat capacity, *C_w_* (J·kg^-1^·K^-1^)	4182	[25]
Thermal conductivity, *k_w_* (W·m^-1^·K^-1^)	0.6	[25]
**At the GNPs/water interface**		
Thermal conductivity, *G* (W·m^-2^·K^-1^)	105 × 10^6^	[25]

## Supplementary Materials

The following are available online at https://www.mdpi.com/2079-4991/9/5/711/s1, Figure S1: The GNP-water system model.
