# Supplementary material for: Photothermal Response of Hollow Gold Nanorods under Femtosecond Laser Irradiation
Source: Nanomaterials (Basel). 2019 May 7;9(5):711. doi: 10.3390/nano9050711 (PMC6566344; doi:10.3390/nano9050711)
Supplement: Supplementary file 1 [file nanomaterials-09-00711-s001.pdf]

Supporting materials

Photothermal Response of Hollow Gold Nanorods under Femtosecond Laser Irradiation

Rongping Gan[1], Haihua Fan[1], Zhongchao Wei[1], Haiying Liu[1], Sheng Lan[1], and Qiaofeng Dai[1,*]

1 Guangdong Provincial Key Laboratory of Nanophotonic Functional Materials and Devices, School of Information and Optoelectronic Science and Engineering, South China Normal University, Guangzhou 510006, China; z30803080@126.com (R.G.); 20111249@m.scnu.edu.cn (H.F.); weizhongchao@263.net (Z.W.); hyliu@vip.163.com (H.L.); slan@scnu.edu.cn (S.L)

* Correspondence: daiqf@scnu.edu.cn (Q.D.); Tel.: +86-13431013630 (Q.D.)

1. Electromagnetic Interaction Between the GNP-Water System and the Laser Pulse

The electric field distribution $\vec{E}(r,t)$ is calculated by using the Helmholtz equation [1]:

$$\nabla \times \left(\mu_r^{-1} \nabla \times \vec{E} \right) - k_0^2 \left(\varepsilon_r - j \frac{\sigma}{\omega \varepsilon_0} \right) \vec{E} = 0 \quad (1)$$

where k_0 is the wave number and the relative permeability μ_r is set to be unity. σ is the conductivity, and ω is the angular frequency. ε_0 is the vacuum dielectric constant and ε_r is the relative permittivity. For the GNP, ε_r is obtained from Johnson and Christy by interpolation [2]. For water, ε_r is taken as 1.77.

The absorption cross-section is defined as $C_{abs}^{\lambda} = Q_{abs} / I_0$. The energy absorbed by GNP is given by the following formula:

$$Q_{abs} = \frac{1}{2} \mathrm{Re} \left[\iint_s \vec{E}_{tot} \times \vec{H}_{tot} \cdot \vec{n}\, ds \right] \quad (2)$$

where $\vec{n}$ represents an outward unit vector normal to the surface of the GNP. $\vec{E}$ and $\vec{H}$ are the electric and magnetic field vectors, respectively. $I_0 = (1/2)\varepsilon_0 n_w E_0^2$ is the intensity of the incident laser, E_0 is the amplitude, and n_w is the refractive index of water.

2. The Preparation of the Starting System

In COMSOL model, the constructed GNP-water system is shown in Figure S1. The heat source of this system is the energy absorbed by GNP *S(t)*, which acts on the domain represented by GNP. The expression *S(t)* can be written as:

$$S(t) = \frac{C_{abs}^{\lambda} \cdot P(t)}{V_p} \quad (3)$$

$$P(t) = \frac{F_L}{\sqrt{2\pi}\, t_\sigma} \exp\left(\frac{-[t - t_0]^2}{2 t_\sigma^2} \right) \quad (4)$$

where C_{abs}^{λ} is the absorption cross section of the GNP and V_p is the volume of the GNP. *P(t)* is the intensity of the pulsed laser with a Gaussian distribution, and F_L is the energy density of the incident laser. $t_\sigma = t_l / 2\sqrt{2 \ln 2}$, in which is the laser pulse width (the full width at half maximum of the Gaussian temporal profile) and t_0 is the position of the center of the peak.

The computation domain is 400 × 400 × 400 nm. The mesh division of the model uses a free tetrahedral mesh with a minimum mesh size of 1 nm. The configuration parameters of our

computer: Intel (R) Xeon (R) CPU E5-1650 0 @ 3.20 GHz, RAM 32.0 GB.

The initial and boundary conditions are set as follows: (i) Initially, the temperature of electrons, lattice, and the surrounding medium are set to 300 K. In other words, at the initial moment, they are at thermal equilibrium. (ii) In the process of the heat transfer, a temperature jump appears at the interface between the surrounding medium and the GNP. Accordingly, the interface thermal conductivity G is used to describe the energy exchange between them. (iii) The boundaries far away from the GNP are set to Dirichlet boundary conditions with a temperature of 300 K.

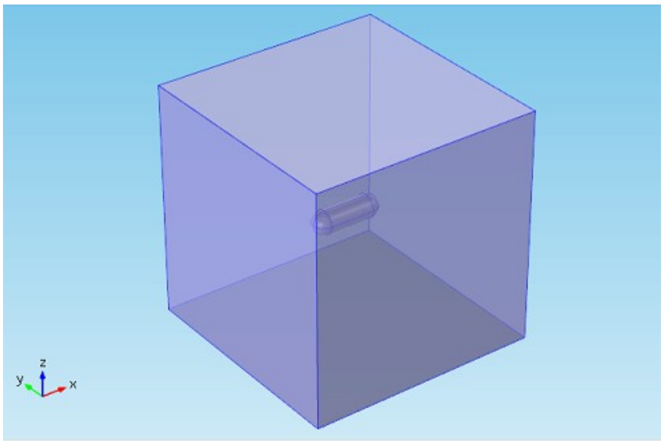

Figure S1. The GNP-water system model.

References

1. Furlani, E. P.; Karampelas, I. H.; Xie, Q. Analysis of pulsed laser plasmon-assisted photothermal heating and bubble generation at the nanoscale. *Lab on a Chip*, **2012**, *12*, 3707-3719. [CrossRef] [PubMed]
2. Johnson, P.B.; Christy, R.W. Optical Constants of the Noble Metals. *PHYSICAL REVIEW B* **1972**, *6*, 4370-4379. [CrossRef] [PubMed]
